# Association Behavior of Amphiphilic ABA Triblock Copolymer Composed of Poly(2-methoxyethyl acrylate) (A) and Poly(ethylene oxide) (B) in Aqueous Solution

**DOI:** 10.3390/polym14091678

**Published:** 2022-04-20

**Authors:** Yoko Mizoue, Ema Onodera, Kazutoshi Haraguchi, Shin-ichi Yusa

**Affiliations:** 1 Department of Applied Chemistry, Graduate School of Engineering, University of Hyogo, 2167 Shosha, Himeji 671-2280, Hyogo, Japan; ym85725@gmail.com (Y.M.); e629ma.o@gmail.com (E.O.); 2 Department of Applied Molecular Chemistry, College of Industrial Technology, Nihon University, 1-2-1 Izumicho, Narashino 275-8575, Chiba, Japan; haraguchi.kazutoshi@nihon-u.ac.jp

**Keywords:** flower-like micelle, triblock copolymer, amphiphilic copolymer, single-electron transfer-living radical polymerization, poly(ethylene oxide)

## Abstract

Poly(2-methoxyethyl acrylate) (PMEA) and poly(ethylene oxide) (PEO) have protein-antifouling properties and blood compatibility. ABA triblock copolymers (PMEA*_l_*-PEO_11340_-PMEA*_m_* (MEOM*_n_*; *n* is average value of *l* and *m*)) were prepared using single-electron transfer-living radical polymerization (SET-LRP) using a bifunctional PEO macroinitiator. Two types of MEOM*_n_* composed of PMEA blocks with degrees of polymerization (DP = *n*) of 85 and 777 were prepared using the same PEO macroinitiator. MEOM*_n_* formed flower micelles with a hydrophobic PMEA (A) core and hydrophilic PEO (B) loop shells in diluted water with a similar appearance to petals. The hydrodynamic radii of MEOM_85_ and MEOM_777_ were 151 and 108 nm, respectively. The PMEA block with a large DP formed a tightly packed core. The aggregation number (*N*_agg_) of the PMEA block in a single flower micelle for MEOM_85_ and MEOM_777_ was 156 and 164, respectively, which were estimated using a light scattering technique. The critical micelle concentrations (CMCs) for MEOM_85_ and MEOM_777_ were 0.01 and 0.002 g/L, respectively, as determined by the light scattering intensity and fluorescence probe techniques. The size, *N*_agg_, and CMC for MEOM_85_ and MEOM_777_ were almost the same independent of hydrophobic DP of the PMEA block.

## 1. Introduction

Amphipathic block copolymers form interpolymer aggregates because of the hydrophobic interactions of hydrophobic blocks in water [1]. Generally, amphipathic AB diblock copolymers form core–shell spherical polymer micelles in water. ABA triblock copolymers with two hydrophobic A blocks at both ends of the central B block form flower-like micelles caused by interpolymer aggregation in water [2]. The hydrophobic A blocks aggregate to form a core, and the hydrophilic B blocks form loop shape shells with a similar appearance to petals on the surface of the core to form flower micelles. The flower micelles are bridged when the hydrophobic A blocks in the ABA triblock copolymer are incorporated into separate cores in flower micelles. With increasing polymer concentration (*C*_p_), the number of bridges between the flower micelles increases to form a gel [3]. The polymers increase the viscosity of the aqueous solution to form interpolymer aggregates, which can then be applied as associative thickeners with a small amount of addition [4]. For example, flower micelles formed from ABA triblock copolymers have been applied as associative thickeners [5]. Associative thickeners are used in water-based paints, coatings, personal care goods, and adhesive agents [6].

The hydrophilic shells on the surface of polymer micelles formed from amphipathic block copolymers stabilize the micelle structure and maintain their dispersion stability in solution [7]. Polymer micelles formed from high molecular weight polymers generally have a lower critical micelle concentration (CMC) and higher colloidal stability than those formed from low molecular weight surfactants [8]. The CMC of amphiphilic diblock copolymers depends on the ratio of the hydrophobic to hydrophilic block lengths. CMC decreases with increasing hydrophobic block chain length for a constant hydrophilic block chain length in the diblock copolymer [9,10,11]. Zhulina et al. [12] studied thermodynamic properties of block copolymer micelles. With ABA triblock copolymers, the CMC also decreases with increasing hydrophobic block chain length [13]. Borisov and Halperin reported theoretical models of flower micelles [14].

Single-electron transfer-living radical polymerization (SET-LRP) is a method for controlled radical polymerization using a copper catalyst [15]. A copper catalyst is widely used as an inorganic electron donor reagent for organic and polymer syntheses (Figure S1). SET-LRP can be performed at low temperatures, e.g., room temperature, because of the low activation energy [16]. Poly(ethylene oxide) (PEO) is often used as a hydrophilic block in amphipathic ABA triblock copolymers because it can form flower micelles easily [17,18]. Furthermore, PEO is widely used in biomedical and biomaterial fields owing to its biocompatibility [19]; 2-Methoxyethyl acrylate (MEA) is an acrylate monomer that can be polymerized by radical polymerization [20]. Poly(2-methoxyethyl acrylate) (PMEA) is highly blood compatible because it has a protein-antifouling effect, and platelets cannot adhere easily to PMEA [21,22]. PMEA forms an intermediate water layer on its surface to suppress protein adsorption [23]. Furthermore, PMEA can be applied as coatings on various substrates because PMEA can be soluble in organic solvents, water insoluble, transparent, and adhesive [24]. Owing to the excellent properties of PMEA, it is also used as a coating material for artificial organs [25]. Haraguchi et al. [26,27] reported protein antifouling and blood compatible coatings using amphiphilic ABA triblock copolymers composed of hydrophobic PMEA (A) and hydrophilic poly(*N,N*-dimethylacrylamide) (B). The hydrophobic PMEA (A) blocks show good adhesion to both organic and inorganic substrates.

**Figure 1:**
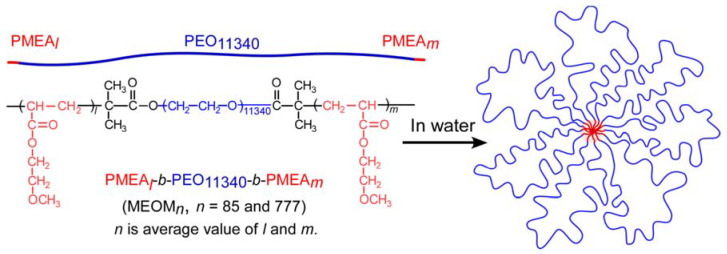
Conceptual illustration of flower micelles formed from MEOM*_n_* (*n* = 85 and 777).

In this study, ABA triblock copolymers (PMEA*_l_*-PEO_11340_-PMEA*_m_* (MEOM*_n_*; *n* is average value of *l* and *m*)) were prepared by SET-LRP to polymerize MEA using a bifunctional PEO macroinitiator at both chain ends. In particular, we are interested in the association behavior of ABA triblock copolymers with long PEO (B) block in water. MEOM*_n_* was composed of a hydrophobic PMEA (A) block and a hydrophilic PEO (B) block. In water, MEOM*_n_* formed flower micelles with a hydrophobic PMEA core and PEO loop-shaped shells (Figure 1). The associative behavior of the flower micelles formed from MEOM*_n_* in dilute aqueous solutions was examined using dynamic light scattering (DLS), static light scattering (SLS), transmission electron microscopy (TEM), and fluorescence probe technique.

## 2. Materials and Methods

### 2.1. General

Tris(2-(dimethylamino)ethyl)amine (Me_6_TREN, 97%) was obtained from Sigma-Aldrich (St. Louis, MO, USA). Copper bromide (CuBr, 95.0%) was supplied by Kishida Chemical (Osaka, Japan). Ethanol (99.5%), tetrahydrofuran (THF, 99.5%), and cetylpyridinium chloride (90%) were purchased from Fujifilm Wako Pure Chemical (Osaka, Japan). All chemicals were used as received; 2-Methoxyethyl acrylate (MEA, 98.0%) from Fujifilm Wako Pure Chemical (Osaka, Japan) was treated with an inhibitor-remover prepacked column from Sigma-Aldrich (St. Louis, MO, USA) prior to use. PEO (MW = 500,000 g/mol) was purchased from Fujifilm Wako Pure Chemical (Osaka, Japan). PEO-based macroinitiator (PEO-Br) was prepared and purified in accordance with the literature [28]. Number average molecular weight (*M*_n_(GPC)) and molecular weight distribution (*M*_w_/*M*_n_) estimated from gel-permeation chromatography (GPC) for PEO-Br were 4.64 × 10^5^ g/mol and 1.23, respectively. The degree of polymerization (DP) for PEO-Br was 11,340. Pyrene (97%) from Fujifilm Wako Pure Chemical (Osaka, Japan) was recrystallized from methanol. Water was purified using an ion-exchange column system.

### 2.2. Preparation of MEOM_n_ (n = 85 and 777)

MEOM_85_ was prepared via SET-LRP (Scheme S1). Me_6_TREN (2.13 mg, 3.01 μmol) was dissolved in water (1.00 mL) and stirred under an argon atmosphere for 10 min. CuBr (2.96 mg, 20.6 μmol) was then added, and the mixture was stirred for 10 min. PEO-Br (*M*_n_(GPC) = 4.64 × 10^5^ g/mol, 1.50 g, 3.01 μmol) and MEA (420 mg, 3.22 mmol) were dissolved in water (21.3 mL). The aqueous CuBr/Me_6_TREN solution was added to an aqueous PEO-Br and MEA solution under an argon atmosphere. The reaction solution was stirred for 71 h under an argon atmosphere at room temperature. The conversion of MEA was 16.8%, which was estimated by ^1^H nuclear magnetic resonance (NMR) spectroscopy before purification. The polymerization mixture was dialyzed against pure water for three days, and the polymer (MEOM_85_) was collected by freeze-drying (0.889 g, 46.3%). The DP of the PMEA block was 85, as estimated from the ^1^H NMR spectrum. The *M*_n_(GPC) and *M*_w_/*M*_n_ estimated from GPC were 5.41 × 10^5^ g/mol and 1.17, respectively.

MEOM_777_ was prepared using the same procedure (2.42 g, 63.7%). The conversion of MEA before purification was 34.9%, according to ^1^H NMR spectroscopy. The DP of the PMEA block was 777, as estimated from the ^1^H NMR spectrum. The *M*_n_(GPC) and *M*_w_/*M*_n_ were 4.91 × 10^5^ g/mol and 1.26, respectively.

### 2.3. Preparation of MEOM_777_ Aqueous Solution

MEOM_777_ (5.86 mg, 8.35 μmol) was dissolved in THF (6.02 mL), and the *C*_p_ was adjusted to 1.00 g/L. The THF solution was dialyzed against pure water for two days to remove THF. After dialysis, the aqueous solution was diluted with water to be *C*_p_ = 0.10 g/L.

### 2.4. Measurements

Using a Bruker (Billerica, MA, USA) DRX-500 and JEOL (Tokyo, Japan) JNM-ECZ400R at 25 °C, ^1^H NMR spectroscopy was performed. The water suppression by gradient-tailored excitation (Watergate) with a double pulse field gradient spin echo pulse sequence was used for the D_2_O solutions to suppress the water signal. Water suppression by a gradient-tailored excitation (WATERGATE) method was used for the D_2_O sample to reduce the water signal. The GPC measurements were conducted at 40 °C using a Shodex (Tokyo, Japan) DS-4 pump, a Shodex GF-7M column, and a Shodex RI-101 refractive index detector. THF was used as the eluent with a flow rate of 1.0 mL/min. *M*_n_(GPC) and *M*_w_/*M*_n_ were determined using standard polystyrene samples. The samples were analyzed by attenuated total reflection-Fourier-transform infrared (ATR-FTIR, FT/IR-4200, Jasco, Tokyo, Japan) spectroscopy. DLS measurements were performed at 25 °C using a Malvern (Malvern, UK) Zetasizer nano ZS at a scattering angle of 173°. The data were analyzed using a Malvern (Malvern, UK) Zetasizer Software package 7.11 to determine the hydrodynamic radius (*R*_h_), light scattering intensity (LSI), and polydispersity (PDI). SLS measurements were taken at 25 °C using an Otsuka Electronics (Osaka, Japan) DLS-7000. The weight-average molecular weight (*M*_w_(SLS)) was calculated from Debye plots. The refractive index increment (d*n*/d*C*_p_) was determined using an Otsuka Electronics (Osaka, Japan) DRM-3000 differential refractometer at 25 °C. Transmission electron microscopy (TEM, JEM-2100, Jeol, Tokyo, Japan) was performed at an acceleration voltage of 160 kV. The TEM samples were prepared by placing a drop of the sample solution on a copper grid coated with a form bar, and the samples were stained with a sodium phosphotungstate aqueous solution. The samples were dried for one day under reduced pressure. Fluorescence measurements were taken using a Hitachi High-Tech (Tokyo, Japan) F-2500 fluorescence spectrophotometer. The pyrene aqueous solutions (6.0 ×10^−7^ M) were excited at 334 nm; the excitation and emission slit widths were 20 and 2.5 nm, respectively.

## 3. Results and Discussion

### 3.1. Characterization

**Figure 2:**
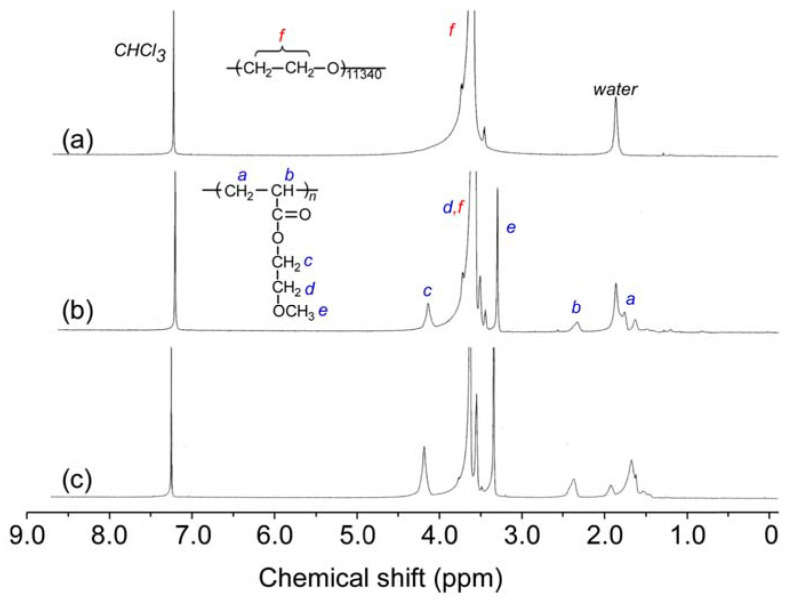
^1^H NMR spectra of (**a**) PEO-Br, (**b**) MEOM_85_, and (**c**) MEOM_777_ in CDCl_3_.

MEOM*_n_* was prepared by SET-LRP using MEA and PEO-Br macroinitiator. The ^1^H NMR spectra for PEO-Br and MEOM*_n_* were measured in CDCl_3_ (Figure 2). The terminal groups in PEO-Br could not be observed clearly because of the low signal intensity. The integral intensities of the PMEA pendant methylene protons at 3.3 ppm (*e*) and the PEO main chain methylene protons at 3.5–4.0 ppm (*f*) were compared to estimate the DP (NMR = *n*) of one end of the PMEA block in MEOM*_n_*. The DP(NMR) values for MEOM_85_ and MEOM_777_ were 85 and 777, respectively (Table 1).

The theoretical DP(theo) and number average molecular weight (*M*_n_(theo)) can be calculated from the following equations:

(1) $$\mathrm{DP}\left(\mathrm{theo}\right)=\frac{{\left[M\right]}_{0}}{{\left[\mathrm{Br}\right]}_{0}}\times \frac{\mathrm{Conversion}\;\left(\%\right)}{100}$$

(2) $$M_{n}\left(\mathrm{theo}\right)=\mathrm{DP}\left(\mathrm{theo}\right)\times M_{n}+M_{\mathrm{PEO}}$$

where [M]_0_ and [Br]_0_ are the initial concentrations of the monomer and bromine atoms at the PEO-Br chain ends, respectively, and *M*_m_ and *M*_PEO_ are the molecular weights of the monomer and PEO, respectively. The DP(theo) values of MEOM_85_ and MEOM_777_ were 86 and 1020, respectively. These theoretical values were close to the DP(NMR) values. GPC was performed for MEOM*_n_* using THF as an eluent (Figure S2). The structure of MEOM*_n_* could be controlled because the *M*_w_/*M*_n_ values estimated from GPC were less than 1.3. However, the retention time for the GPC elution curves of MEOM*_n_* was similar to that of PEO-Br. Unexpected interactions may have occurred between the MEOM*_n_* and GPC column, and polystyrene was used as the standard that may have impeded a correct estimation of the *M*_n_(GPC) [29].

ATR-FTIR was performed to characterize the chemical structure of MEOM*_n_* (Figure S3). The C=O vibration stretching peak was observed at 1700 cm^−1^ for MEOM*_n_*, whereas the peak could be observed for PEO-Br. The peak intensity at 1700 cm^−1^ increased with increasing DP of the PMEA block in MEOM*_n_*. These results confirmed that MEOM*_n_* had been prepared.

### 3.2. Association Behavior of MEOM_n_

**Figure 3:**
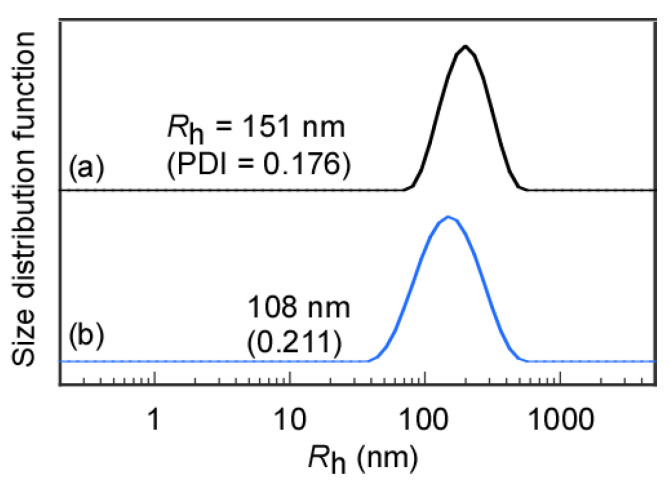
Hydrodynamic radius (*R*_h_) distributions of (**a**) MEOM_85_ and (**b**) MEOM_777_ in water at *C*_p_ = 0.1 g/L.

The *R*_h_ distributions of the flower micelles formed from MEOM*_n_* in water were examined by DLS (Figure 3). MEOM_777_ could not dissolve directly in water because of its long hydrophobic PMEA blocks. Therefore, an aqueous solution was prepared to dialyze the THF solution of MEOM_777_ against water. In contrast, MEOM_85_ could dissolve directly in water. The *R*_h_ values of flower micelles obtained after directly dissolving them in water and after the dialysis method were compared to confirm the difference between the preparation methods of the MEOM_85_ aqueous solutions (Figure S4). The *R*_h_ values for MEOM_85_ prepared by direct dissolution in water and the dialysis method were 151 and 144 nm, respectively, which are similar. Therefore, flower micelles formed from MEOM_85_ regardless of the solution preparation method. The association state of MEOM_85_ that was easily soluble in water reaches the lowest association energy independently of the dissolution methods. Unless noted otherwise, the MEOM_85_ aqueous solution was prepared using the direct dissolution method. The *R*_h_ distributions for MEOM*_n_* in water were unimodal. The *R*_h_ values for MEOM_85_ and MEOM_777_ were 144 and 108 nm, respectively. The DP of the hydrophobic PMEA block in MEOM_777_ was larger than that in MEOM_85_, but *R*_h_ of MEOM_777_ was smaller than that of MEOM_85_. As the DP of the PMEA block in MEOM*_n_* increased, the hydrophobic interaction became stronger to form a more compact core of the flower micelle. The PDI values for MEOM_85_ and MEOM_777_ were 0.166 and 0.211, respectively. MEOM_85_ formed more uniformly sized flower micelles than MEOM_777_.

The structure of flower micelles formed from MEOM*_n_* in water was confirmed by SLS (Figure S5). The apparent weight-average molecular weight (*M*_w_(SLS)) and radius of gyration (*R*_g_) were obtained from the SLS measurements. The refractive index increment (d*n*/d*C*_p_) required to determine *M*_w_(SLS) was obtained using a differential refractometer. The d*n*/d*C*_p_ values for MEOM_85_ and MEOM_777_ were 0.138 and 0.426 mL/g, respectively. The number of PMEA chains (*N*_agg_) forming a single flower micelle was calculated from the equation, *N*_agg_ = 2*M*_w_(SLS)/(*M*_n_(NMR) × *M*_w_/*M*_n_). The *N*_agg_ values for MEOM_85_ and MEOM_777_ were 156 and 164, respectively (Table 2). The DP of the PMEA block in MEOM_777_ was approximately nine times larger than that in MEOM_85_, but both *N*_agg_ values were close. The interface between the core and shell was sterically crowded with the PEO chains because the DP of the PEO block forms a loop-shaped shell. It was unlikely that the *N*_agg_ value would increase above a certain number because of the congestion of the PEO shell chains on the core–shell interface. The *R*_g_ values for MEOM_85_ and MEOM_777_ were 141 and 164 nm, respectively. From the *R*_h_ and *R*_g_ values, the flower micelles formed from MEOM_85_ and MEOM_777_ have similar size. The *R*_g_/*R*_h_ ratios for MEOM_85_ and MEOM_777_ were 0.934 and 1.52, respectively. These *R*_g_/*R*_h_ ratios were close to one, suggesting that the shape of flower micelles was spherical [30]. The density (Φ_H_) of the micelle can be calculated from Equation (3) [31]:

(3) $$\Phi_{H}=\frac{M_{w}\left(\mathrm{SLS}\right)}{N_{A}}{\left(\frac{4}{3}\pi R_{h}{}^{3}\right)}^{-1}$$

where *N*_A_ is Avogadro’s number. The Φ_H_ values for MEOM_85_ and MEOM_777_ were 5.47 × 10^−3^ and 2.29 × 10^−2^ g/mL, respectively. MEOM_777_ with a long PMEA chain formed a tightly packed core because the Φ_H_ value of MEOM_777_ was larger than that of MEOM_85_; ^1^H NMR spectroscopy of MEOM*_n_* was performed in D_2_O (Figure S6). The PEO signals were observed, but the PMEA signals were not. This observation suggests that the motion of PMEA was restricted due to the formation of the core, but the motion of PEO was not restricted.

**Figure 4:**
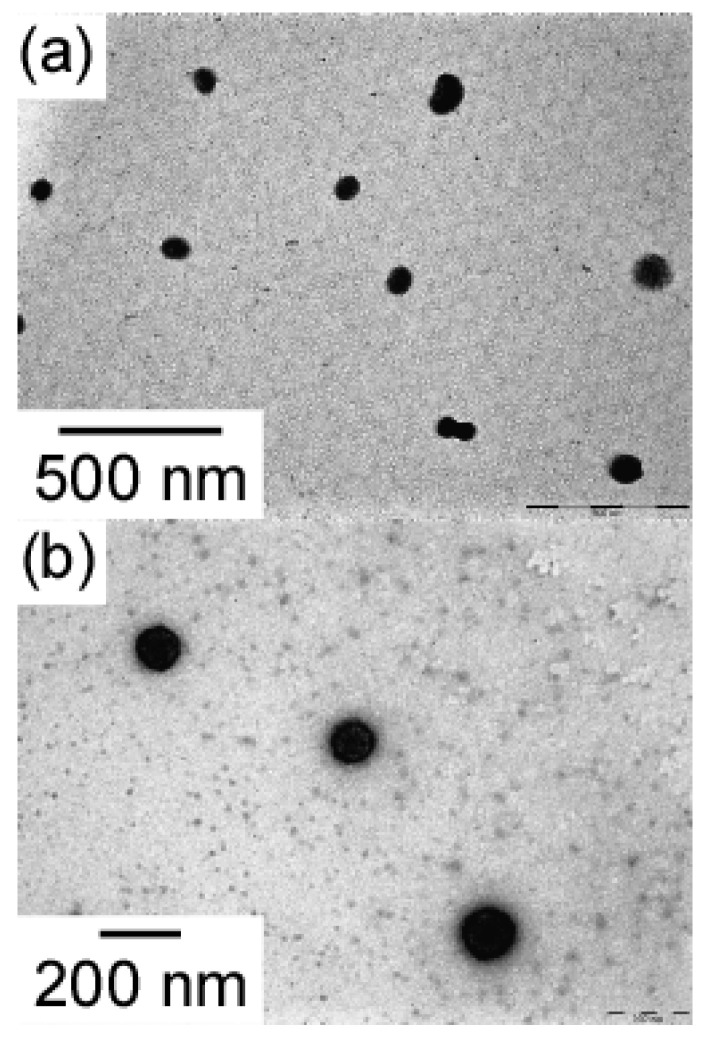
TEM images for (**a**) MEOM_85_ and (**b**) MEOM_777_ in water at *C*_p_ = 0.1 g/L.

TEM of MEOM_85_ and MEOM_777_ in water (Figure 4) revealed spherical aggregates. The radii (*R*_TEM_) of MEOM_85_ and MEOM_777_ estimated from TEM were 42.4 and 59.2 nm, respectively. These *R*_TEM_ values were smaller than *R*_h_ and *R*_g_ obtained from light scattering measurements. The shells formed from PEO were not observed because PEO cannot be stained by sodium phosphotungstate. The core formed by the association of the PMEA blocks could be stained, as observed by TEM. Therefore, the cores observed by TEM were separated a certain distance due to the unstained PEO loop shells that cannot be observed by TEM.

### 3.3. Critical Micelle Concentration (CMC) of MEOM_n_

**Figure 5:**
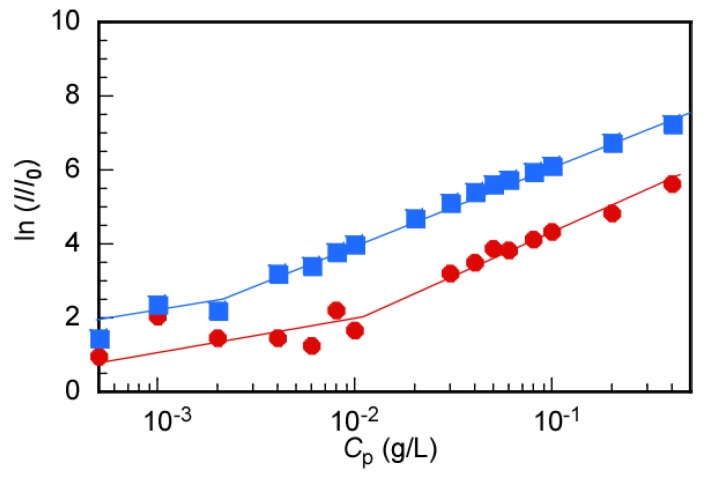
Light scattering intensity (LSI) ratio (*I*/*I*_0_) as a function of polymer concentration (*C*_p_) for MEOM_85_ (●) and MEOM_777_ (■) in aqueous solutions; *I*_0_ is LSI of water, and *I* is LSI of the polymer solution.

To determine the CMC of flower micelles, the LSI for MEOM*_n_* aqueous solutions was measured as a function of *C*_p_ (Figure 5). The ratio (*I*/*I*_0_) of the LSI of the solution (*I*) to the solvent (*I*_0_) was plotted as a function of *C*_p_. The CMC was estimated from the inflection point of the slope [32]. The CMC values for MEOM_85_ and MEOM_777_ were calculated to be 0.01 and 0.002 g/L, respectively (Table 3).

**Figure 6:**
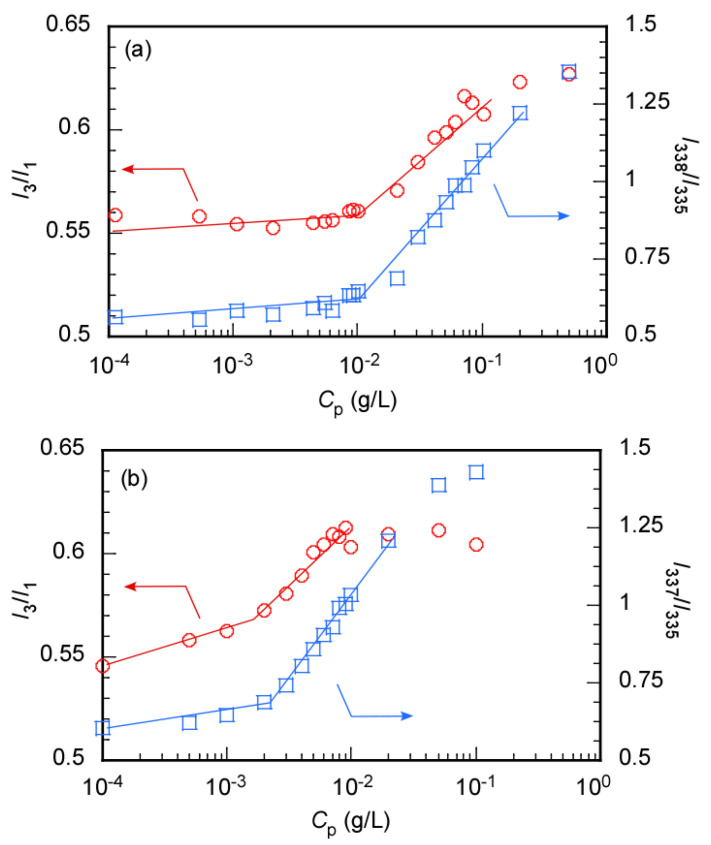
Pyrene fluorescence intensity ratio (*I*_3_/*I*_1_, 〇) and excitation intensity ratio (*I*_338_/*I*_335_, □) as a function of the polymer concentration (*C*_p_) for (**a**) MEOM_85_ and (**b**) MEOM_777_ in aqueous solutions; *I*_3_ and *I*_1_ are the third and first vibronic peak intensities of pyrene fluoresce, and *I*_338_ and *I*_335_ are the peak intensities at 338 and 335 nm in the excitation spectra of pyrene.

The CMC of MEOM*_n_* was also estimated using pyrene as a hydrophobic fluorescence probe (Figure 6). The intensity ratio (*I*_3_/*I*_1_) of the first (*I*_1_) to third vibronic peak (*I*_3_) of the pyrene fluorescence spectrum depends on the microenvironmental polarity around the pyrene molecule [33]. *I*_3_/*I*_1_ increased with decreasing microenvironmental polarity. *I*_3_/*I*_1_ was plotted as a function of *C*_p_ to determine the CMC (CMC(Em)) (Figure 6). CMC(Em) was calculated from the intersections of the two tangents of the plots. The CMC(Em) values for MEOM_85_ and MEOM_777_ were 0.01 and 0.0015 g/L, respectively. The emission maximum wavelength of the 0–0 band in the pyrene excitation spectrum shifts to a longer wavelength when the microenvironment around the pyrene molecule becomes hydrophobic [34]. The 0–0 band maximum wavelengths of the aqueous solutions in the presence and absence of MEOM*_n_* were 338 and 335 nm, respectively. The CMC (CMC(Ex)) was determined from a plot of *I*_338_/*I*_335_ vs. *C*_p_, where *I*_338_ and *I*_335_ are the emission intensities at 338 and 335 nm, respectively. The CMC(Ex) values for MEOM_85_ and MEOM_777_ were 0.01 and 0.002 g/L, respectively. The CMC values estimated from the LSI and fluorescence probe methods were similar. These observations suggest that hydrophobic anticancer drugs can be encapsulated in the core above the CMC.

## 4. Conclusions

Amphiphilic ABA triblock copolymers, MEOM*_n_*, were prepared via SET-LRP using a bifunctional PEO-Br macroinitiator. MEOM_85_ and MEOM_777_ were prepared with different DP of the hydrophobic PMEA blocks at the central PEO chain ends. The DP of the PMEA block in MEOM_777_ was approximately nine times larger than that of MEOM_85_. The *R*_h_ values for flower micelles formed from MEOM_85_ and MEOM_777_ in water were 151 and 108 nm, respectively. The hydrophobic PMEA blocks with a large DP in MEOM_777_ associated to form a densely packed core due to the strong hydrophobic interactions. The *N*_agg_ values for flower micelles formed from MEOM_85_ and MEOM_777_ were similar. The CMC for MEOM_85_ and MEOM_777_ were 0.01 and 0.002 g/L, respectively. The CMC of MEOM_777_ was smaller than that of MEOM_85_ because of the strong hydrophobic interactions of MEOM_777_. These results may come from much larger DP of the PEO block than that of the PMEA blocks. The PEO blocks that formed the loop shells of flower micelles and the PMEA blocks that formed the core were both biocompatible. Therefore, the biocompatible flower micelles formed from MEOM*_n_* may have applications as novel drug delivery carriers. We believe that the chemical design of MEOM*_n_* can be applied for coating on the various biomedical devices.

## Figures and Tables

**Table 1:** Characteristics of polymers.

Sample	*M*_n_(Theo) ×10^5^	DP(Theo)	*M*_n_(NMR) ×10^5^	DP(NMR)	*M*_n_(GPC) ×10^5^	*M*_w_/*M*_n_ (GPC)
PEO-Br	5.00 *^a^*	11,340 *^a^*	5.00	-	4.64	1.23
MEOM_85_	5.11	86 *^b^*	5.22	85 *^b^*	5.41	1.17
MEOM_777_	7.65	1020 *^b^*	7.02	777 *^b^*	4.91	1.26

*^a^* These values of PEO base material were provided by the supplier. *^b^* Degree of polymerization of one end of PMEA.

**Table 2:** Characteristics of MEOM*_n_* flower-like micelles in water.

Sample	*M*_w_(SLS) ^a^ × 10^−7^ (g/mol)	*R*_g_ ^a^ (nm)	*R*_h_ ^b^ (nm)	*R*_g_/*R*_h_	*R*_TEM_^c^ (nm)	*N*_agg_ (SLS) ^d^	Φ_H_ × 10^2^ (g/mL)
MEOM_85_	4.75	141	151	0.934	42.4	156	0.547
MEOM_777_	7.27	164	108	1.52	59.2	164	2.29

^a^ Estimated from SLS measurements; ^b^ estimated from DLS measurements; ^c^ estimated from TEM; ^d^ calculated from 2*M*_w_(SLS)/(*M*_n_(NMR) × *M*_w_/*M*_n_).

**Table 3:** Critical micelle concentration (CMC) of MEOM*_n_*.

Sample	CMC(LSI) (g/L)	CMC(Em) (g/L)	CMC(Ex) (g/L)
MEOM_85_	0.01	0.01	0.01
MEOM_777_	0.002	0.0015	0.002

## Data Availability

Not applicable.

## Supplementary Materials

The following are available online at www.mdpi.com/article/10.3390/polym14091678/s1, Scheme S1. Synthesis of MEOM*_n_* (*n* = 85 and 777); Figure S1. Mechanism of single-electron transfer-living radical polymerization (SET-LRP); M; monomer, P; polymer, Cu; copper, X; halogen, L; ligand, kact; activation, kdeact; deactivation, kp; propagation, kt; termination; Figure S2. Gel-permeation chromatography (GPC) elution curves of PEO11340-Br (black line), MEOM85 (red line), and MEOM777 (blue line) using THF as an eluent with a flow rate of 1.0 mL/min at 40 °C; Figure S3. Attenuated total reflection (ATR) Fourier-transform infrared (FTIR) spectra for (a) PEO11340-Br, (b) MEOM85, and (c) MEOM777; Figure S4. Hydrodynamic radius (Rh) distributions of MEOM85 aqueous solution prepared by directly dissolution (blue line) and by dialysis from THF solution against aqueous solution (red line); the final polymer concentration (Cp) was adjusted to 0.1 g/L; Figure S5. Debye plots for (a) MEOM85 and (b) MEOM777 in water; Figure S6. Water suppression by gradient-tailored excitation (WATERGATE) ^1^H NMR spectroscopy in D_2_O for (a) MEOM_85_ and (b) MEOM_777_ at 25 °C.
