# Supplementary material for: Association Behavior of Amphiphilic ABA Triblock Copolymer Composed of Poly(2-methoxyethyl acrylate) (A) and Poly(ethylene oxide) (B) in Aqueous Solution
Source: Polymers (Basel). 2022 Apr 20;14(9):1678. doi: 10.3390/polym14091678 (PMC9105209; doi:10.3390/polym14091678)
Supplement: Supplementary file 1 [file polymers-14-01678-s001.zip › polymers-1663206-supplementary.pdf]

SUPPORTING INFORMATION

Association Behavior of Amphiphilic ABA Triblock Copolymer Composed of Poly(2-methoxyethyl acrylate) (A) and Poly(ethylene oxide) (B) in Aqueous Solution

Yoko Mizoue[1], Ema Onodera[1], Kazutoshi Haraguchi[2], Shin-ichi Yusa[1,*]

[1]Department of Applied Chemistry, Graduate School of Engineering, University of Hyogo, 2167 Shosha, Himeji, Hyogo 671-2280, Japan; ym85725@gmail.com

[2]College of Industrial Technology, Nihon University, 1-2-1 Izumicho, Narashino, Chiba, Japan; haraguchi.kazutoshi@nihon-u.ac.jp

*Correspondence: yusa@eng.u-hyogo.ac.jp

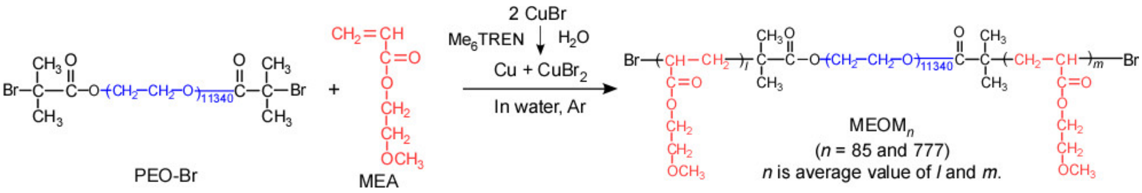

Scheme S1. Synthesis of MEOM$_n$ (n = 85 and 777).

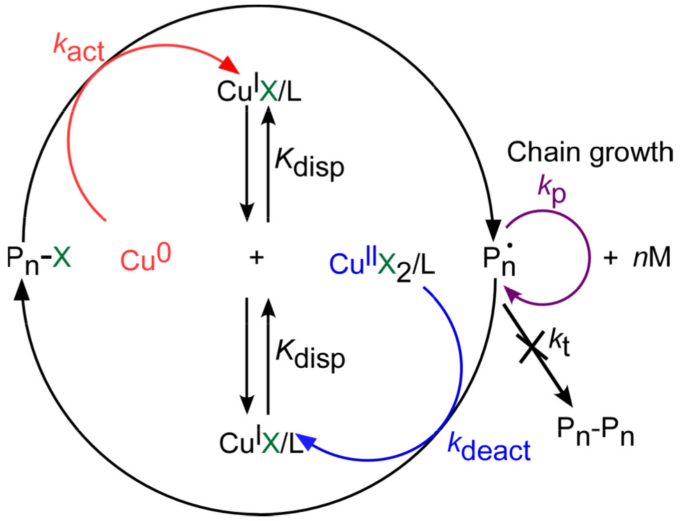

Figure S1. Mechanism of single-electron transfer-living radical polymerization (SET-LRP); M; monomer, P; polymer, Cu; copper, X; halogen, L; ligand, k_{act}; activation, k_{deact}; deactivation, k_p; propagation, k_t; termination.

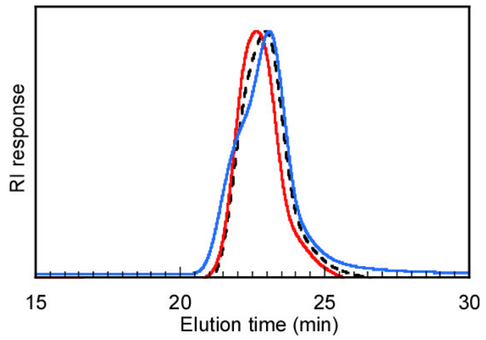

Figure S2. Gel-permeation chromatography (GPC) elution curves of PEO_{11340}-Br (black line), $MEOM_{85}$ (red line), and $MEOM_{777}$ (blue line) using THF as an eluent with a flow rate of 1.0 mL/min at 40 °C.

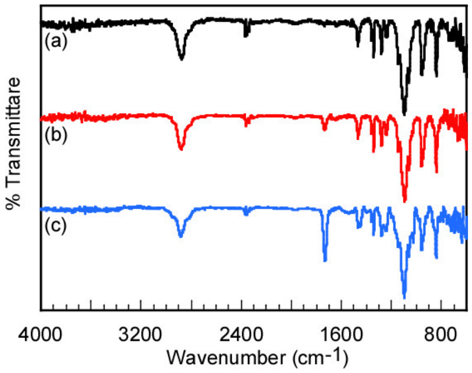

Figure S3. Attenuated total reflection (ATR) Fourier-transform infrared (FTIR) spectra of (a) PEO_{11340}-Br, (b) $MEOM_{85}$, and (c) $MEOM_{777}$.

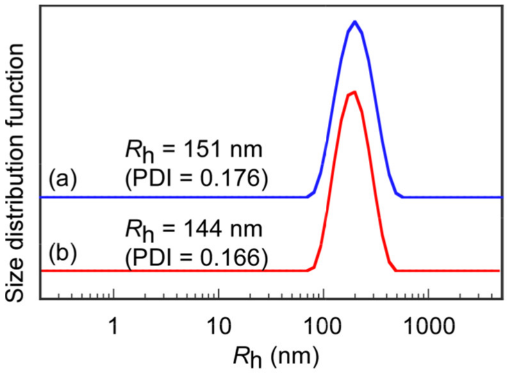

Figure S4. Hydrodynamic radius (R_h) distributions of $MEOM_{85}$ aqueous solution prepared by directly dissolution (blue line) and by dialysis from THF solution against aqueous solution (red line); the final polymer concentration (C_p) was adjusted to 0.1 g/L.

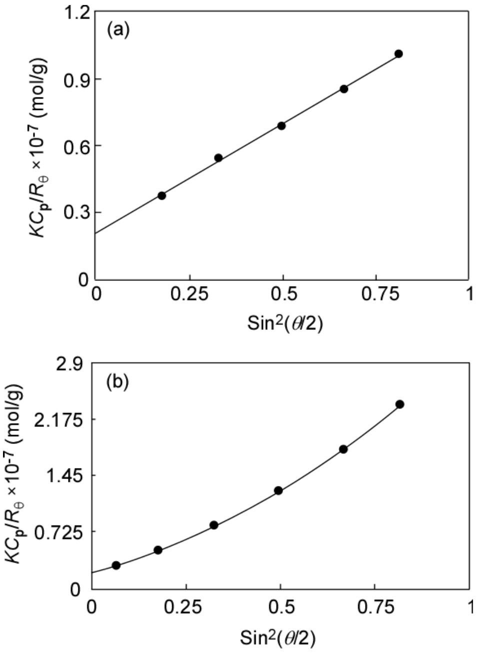

Figure S5. Debye plots for (a) $MEOM_{85}$ and (b) $MEOM_{777}$ in water.

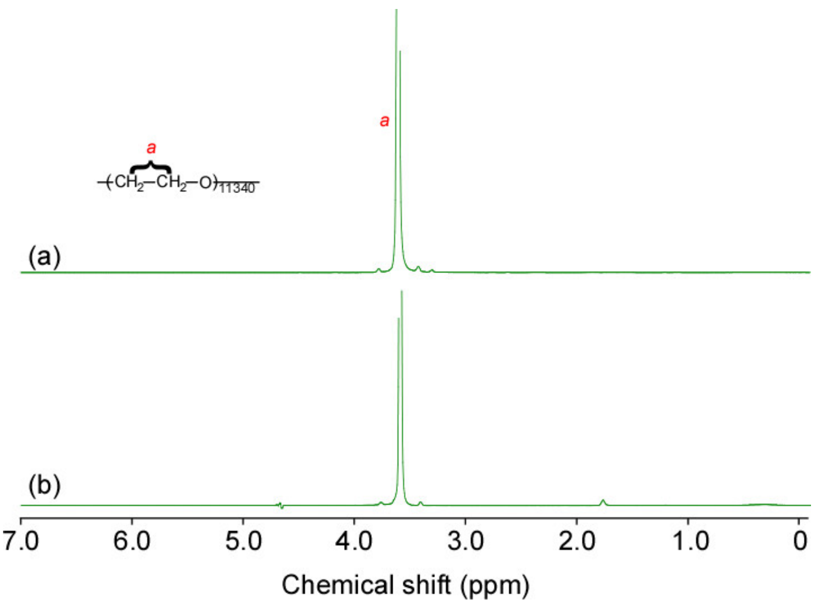

Figure S6. Water suppression by gradient-tailored excitation (WATERGATE) ^1H NMR spectroscopy in D_2O for (a) $MEOM_{85}$ and (b) $MEOM_{777}$ at 25 °C.
